# Holding Thermal Receipt Paper and Eating Food after Using Hand Sanitizer Results in High Serum Bioactive and Urine Total Levels of Bisphenol A (BPA)

**DOI:** 10.1371/journal.pone.0110509

**Published:** 2014-10-22

**Authors:** Annette M. Hormann, Frederick S. vom Saal, Susan C. Nagel, Richard W. Stahlhut, Carol L. Moyer, Mark R. Ellersieck, Wade V. Welshons, Pierre-Louis Toutain, Julia A. Taylor

**Affiliations:** 1 Division of Biological Sciences, University of Missouri, Columbia, Missouri, United States of America; 2 Department of Obstetrics, Gynecology and Women’s Health, University of Missouri, Columbia, Missouri, United States of America; 3 Department of Statistics, University of Missouri, Columbia, Missouri, United States of America; 4 Department of Biomedical Sciences, University of Missouri, Columbia, Missouri, United States of America; 5 Université de Toulouse, INPT, ENVT, UPS, UMR1331, F- 31062 Toulouse, France; 6 INRA, UMR1331, Toxalim, Research Centre in Food Toxicology, F-31027 Toulouse, France; Institute for Health & the Environment, United States of America

## Abstract

Bisphenol A (BPA) is an endocrine disrupting environmental contaminant used in a wide variety of products, and BPA metabolites are found in almost everyone’s urine, suggesting widespread exposure from multiple sources. Regulatory agencies estimate that virtually all BPA exposure is from food and beverage packaging. However, free BPA is applied to the outer layer of thermal receipt paper present in very high (∼20 mg BPA/g paper) quantities as a print developer. Not taken into account when considering thermal paper as a source of BPA exposure is that some commonly used hand sanitizers, as well as other skin care products, contain mixtures of dermal penetration enhancing chemicals that can increase by up to 100 fold the dermal absorption of lipophilic compounds such as BPA. We found that when men and women held thermal receipt paper immediately after using a hand sanitizer with penetration enhancing chemicals, significant free BPA was transferred to their hands and then to French fries that were eaten, and the combination of dermal and oral BPA absorption led to a rapid and dramatic average maximum increase (Cmax) in unconjugated (bioactive) BPA of ∼7 ng/mL in serum and ∼20 µg total BPA/g creatinine in urine within 90 min. The default method used by regulatory agencies to test for hazards posed by chemicals is intra-gastric gavage. For BPA this approach results in less than 1% of the administered dose being bioavailable in blood. It also ignores dermal absorption as well as sublingual absorption in the mouth that both bypass first-pass liver metabolism. The elevated levels of BPA that we observed due to holding thermal paper after using a product containing dermal penetration enhancing chemicals have been related to an increased risk for a wide range of developmental abnormalities as well as diseases in adults.

## Introduction

**Figure 1:**
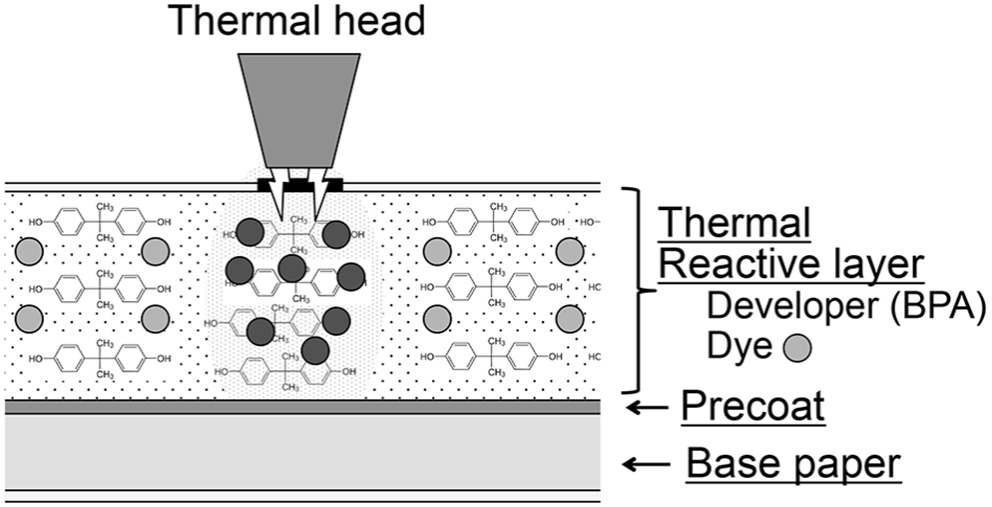
Schematic diagram of thermal receipt paper identifying the thermal reactive layer that contains BPA as a developer and a leuco dye, as well as stabilizers and binders (not shown).

Bisphenol A [BPA; bis(4-hydroxyphenyl)propane; CAS #80-05-7] is one of the highest volume chemicals in commerce with 15-billion pounds produced per year [1], and based on the presence of BPA metabolites in urine, it can be concluded that virtually everyone is exposed [2], [3]. BPA has estrogenic and other endocrine disrupting activities [4], [5]. BPA molecules are polymerized to make polycarbonate plastic used for food and beverage containers, epoxy resins used to line cans, and dental composites and sealants, but free (unpolymerized) BPA is also used as an additive (plasticizer), such as in polyvinyl chloride (PVC) products. Our interest is in the use of BPA in thermal paper, which is used for airline ticket, gas, ATM, cash register and other types of receipts (Figure 1). The print surface of thermal paper is coated with milligrams of free BPA per gram paper as a heat-activated print developer [6], and it appears that free BPA is readily transferred to other materials that the thermal paper contacts [7].

While small lipophilic compounds such as BPA (logP = 3.4; molecular weight 228 Da) can pass through skin [8], [9], regulatory agencies have assumed that this route of human BPA exposure should not be significant in spite of the lack of data and acknowledged “significant uncertainties” around the issue of human exposure to BPA from thermal paper [10]. However, a factor that has not been considered in estimating transdermal exposure to BPA from thermal paper is that hand sanitizers are now commonly used, particularly in fast-food restaurants where people may handle thermal receipts before eating or ordering food. Hand sanitizer and other skin care products may also be used by cashiers while working. Exposure to BPA from thermal paper goes beyond just transdermal exposure and consumption of food that is picked up and eaten with a BPA-contaminated hand. The transfer of a chemical directly from hand-to-mouth (mouthing behavior) has been proposed to be an important variable for estimating total chemical exposure in humans [11], particularly in young children [12].

The use of hand sanitizers and other skin-care products, including soaps, lotions and sunscreens, is significant because some contain mixtures of chemicals that are also used as dermal penetration enhancers to increase the transdermal delivery of drugs. Drugs and chemicals that are suitable for transdermal delivery and are impacted by dermal penetration enhancers have a LogP>1.5 and a molecular weight <500 Da [9]. There are many factors that impact the ability of compounds to pass through skin in addition to molecular weight and lipophilicity, including differences arising from the location of skin on the body, gender and age [13]. Mixtures of dermal penetration enhancing chemicals can act synergistically to increase by up to 100 fold the dermal penetration of small lipophilic molecules such as estradiol [8], [9], with which BPA shares physical-chemical and biological properties [4]. For example, Purell hand sanitizer (Gojo Industries), which we used in the current study, contains a number of dermal penetration enhancers, such as isopropyl myristate and propylene glycol, and is (63% w/w) ethanol. The use of hand sanitizers has increased in recent years and is now about a 200 million dollar a year industry just in the USA [14]. The impact of the use of personal care products such as moisturizing lotions that contain dermal penetration enhancing chemicals on exposure to environmental chemicals has been identified as a concern [15].

To assess the relevance of this research to real-world behavior, we conducted a preliminary observational study in fast-food restaurants, food courts and shopping malls in Columbia Missouri. Receipt contact time varied widely, but was sometimes substantial. In one restaurant, we found that receipt contact time ranged up to 65 sec for people purchasing food that was eaten in the restaurant; the 75th percentile for time holding the receipt was >12 sec, and the 90th percentile >32 sec. In a fast-food restaurant that is part of an international chain, take-out food was placed into a bag and the top of the bag was folded, then the thermal receipt was stapled to the top of the bag; the result was that the print surface of the receipt (coated with BPA) was grabbed when the bag was picked up. The contact time between the hand and thermal receipt was thus considerably longer than would be the case for food eaten in the restaurant. In a food court we observed that some fast-food restaurants had hand sanitizer dispensers available for use by customers next to the cash register, and customers were observed using the hand sanitizer before handling the thermal receipt. The estimate is that 50 million people eat in a fast-food establishment every day in the USA [16]. Finally, our experiments here are also relevant to occupational exposures, because we observed in a national chain big-box store that all cash registers had a hand sanitizer dispenser next to them for use by the cashiers.

Our objectives were to examine the impact of having dry hands vs. wet hands due to using a popular hand sanitizer that contains dermal penetration enhancing chemicals on extraction of BPA from the surface of thermal receipt paper coated with BPA. We also measured (using a LC/MSMS assay) unconjugated, bioactive BPA (uBPA) and its conjugated metabolites, BPA-glucuronide (BPA-G) and BPA-monosulfate (BPA-S), in serum and urine in adult male and female subjects after holding a thermal receipt. To determine the proportion of thermal receipts that contained BPA, we examined receipt papers for the presence and amount of BPA. We also examined receipts for the most commonly used BPA replacement chemical, bisphenol S [bis(4-hydroxyphenyl)sulfone; BPS; CAS #80-09-1].

## Methods

### Ethics statement

The University of Missouri School of Medicine Institutional Review Board approved all procedures involving human subjects, and sample collection was conducted by licensed personnel in the Clinical Research Center (CRC) within the University of Missouri School of Medicine. Subjects were informed of the procedures, and provided written consent. The signed consent forms were retained. The University IRB approved the consent procedure.

### Subjects

Participants for the different experiments in this study were recruited through a weekly University of Missouri campus-wide email newsletter. Candidates (men and women) were pre-screened by age, height, weight, and health status. Participants selected were 20–40 years old (average 27.0 yrs), and an attempt was made to select those with average height, weight and normal-range body-mass index. Participants selected were not taking any prescription or non-prescription medication other than oral contraceptives; the type of oral contraceptive used was recorded. To ensure that pregnant women were excluded from the study, all women were administered a pregnancy test when they arrived at the CRC.

For all studies participants were asked to refrain from touching thermal paper receipts, consuming food or beverages stored in polycarbonate or other types of plastic containers as well as canned food and beverages during the 48 hr prior to participating in the study, in order to reduce background BPA levels in body fluids as much as possible. The participants also filled out a questionnaire concerning their activities during the prior 48 hr (see Section S3 in File S1 for questionnaire).

For experiments in which there was hand contact with thermal receipt paper, subjects were required to wash their hands with soap and water, rinse thoroughly, and then dry using Kimwipes (Kimberly-Clark, Irving, TX). A number of soaps were screened for BPA content and/or chromatographic interference prior to the start of the study, and the soap chosen was Softsoap “Aquarium series” (Colgate Palmolive Company, Manhattan, NY), which showed no detectable BPA or chromatographic interference with the assay of BPA. Standard brown laboratory paper towels were tested and found to contain BPA at around 6 µg/towel. Because of this, Kimwipes, which tested negative for BPA, were used throughout for drying hands. Water from faucets used in the CRC was tested and found to be below the limit of detection (LOD) for BPA content (detection limit was 10 pg/mL by HPLC with CoulArray detection based on C-18 extraction of 250 ml of water).

### Sample analysis

Analysis of BPA in extracted samples occurred within an accredited facility (Veterinary Medicine Diagnostic Laboratory) within the College of Veterinary Medicine at the University of Missouri.

#### Reagents

Solvents (methanol, acetonitrile) and water were HPLC grade, and were obtained from Fisher Scientific. BPA, bisphenol S (BPS), and BPA monosulfate (BPA-S) were obtained from Sigma-Aldrich (St. Louis MO; purity >99%, 98% and 95% respectively). C^13^-BPA was obtained from Cambridge Isotope Laboratories Inc. (Andover, MA; purity 99%), and both BPA-G (purity 98%) and BPA D-glucuronide (BPA-DG; purity >99%) were provided by the National Institute of Environmental Health Sciences (NIEHS), Research Triangle Park, NC. Ethanol (200 proof) used for hand swipes was obtained from Decon Labs, Inc. (King of Prussia, PA).

#### Total receipt BPA and BPS content

Weighed samples of each receipt (3×3 cm) were incubated overnight in methanol at room temperature. The methanol extracts were diluted in methanol, typically to a final dilution of 1/10,000, and BPA content was analyzed by HPLC with CoulArray detection (see Section S1 in File S1 for details). We also analyzed the same receipt sample extracts for BPS using LC/MSMS (see Section S1 in File S1 for details).

#### BPA levels in Kimwipe hand swipes

Kimwipe swipes were incubated in methanol at room temperature overnight, and aliquots were taken from the methanol extract for analysis. BPA in the methanol extract was determined by HPLC with CoulArray detection.

#### BPA levels in French fries

French fries were incubated individually in methanol overnight. The fries were then removed, and the samples centrifuged briefly to separate any solid and/or oily matter, and a sample of the clear methanol extract was assayed. Equal volumes from the 10 extracts from the 10 French fries touched by each participant were pooled, and a single measurement was made for each participant. Quantitation was made by HPLC with CoulArray detection.

#### Serum sample collection and extraction

Multiple-point blood samples were collected via IV catheter into 10 mL syringes, and the syringes were emptied into the same uncoated vacutainer tubes (for details and catalog numbers of collection materials Section S1 in File S1). Single point blood samples were collected by venipuncture into uncoated glass vacutainer tubes (Becton Dickinson, Franklin Lakes, NJ). All blood samples were allowed to clot at room temperature for 15–30 min and then refrigerated until centrifugation at 4°C for 15 min. The serum was transferred with glass Pasteur pipets into 15 mL centrifuge tubes and then frozen at −20°C. Samples were extracted using C18 SPE as previously described [17]; see Section S1 in File S1. Procedural blanks were also run alongside the samples to monitor for reagent contamination or interference. Serum extracts were analyzed by LC/MSMS.

#### Urine sample collection and extraction

All urine samples were collected directly into Samco polypropylene specimen cups (Fisher Scientific, Waltham, MA) and were immediately refrigerated (4°C for 2–5 hours) until they could be transferred to the research laboratory, at which point they were frozen at −20°C. The total BPA concentration (representing a combined measure of unconjugated and conjugated BPA) was measured by LC/MSMS (see Section S1 in File S1).

#### Assay of creatinine in urine

To calculate creatinine-corrected urine BPA concentrations, urine creatinine was measured using an ELISA kit (R&D Systems Inc., Minneapolis, MN), according to manufacturer’s instructions. Sensitivity of this assay is 0.02 mg/dL.

#### Field blanks

The possibility of BPA leaching from each piece of equipment used in the collection or processing of samples identified above was determined by passing BPA-free water through all collection equipment, which was then handled and assayed for BPA as described below for the actual samples. All equipment and sample handling was determined to not leach detectable BPA before any sample collections occurred.

### Statistical methods and calculation of pharmacokinetic parameters

For both uBPA and BPA-G, the area under the concentration-time curve (AUC) up to the last measured serum concentration above the LOQ, i.e. AUC (0–90 min), was calculated by using the linear trapezoidal rule. The average AUC (0–90 min) (ng/mL) was calculated by dividing AUC (0–90 min ng/mL)/90 min. Time (Tmax) of maximal plasma BPA concentration (Cmax) was directly obtained from the raw data. Comparisons of men and women were conducted using the Mann-Whitney U test or ANOVA. Statistical significance was set a P<0.05, two-tailed test. All data are presented as mean±SEM.

### Experiment 1: Measurement of BPA and BPS in 50 used thermal receipt papers

The objective of this experiment was to determine the amount of BPA and BPS in thermal receipt paper and to determine the proportion of receipts that contained BPA or BPS, which is the most commonly used BPA replacement chemical. Thermal paper sales receipts were obtained by purchasing items from 41 different vendors in Columbia, MO and from a further 9 vendors in Southern Missouri (50 receipts total). Weighed portions of each paper were extracted and assayed for BPA by HPLC with CoulArray detection and for BPS by LC/MSMS. After screening, an unused roll was obtained from a vendor from which a BPA-positive receipt had been identified. The BPA content of paper from this roll was confirmed prior to being used for testing with human subjects in Experiments 2, 3 and 4.

### Experiment 2: BPA transferred to a hand with and without using hand sanitizer due to holding a thermal receipt for different lengths of time

The objective of this experiment was to determine the amount of BPA extracted by a hand from a standard piece of thermal receipt paper immediately after using Purell hand sanitizer (Experiment 2-A) or with dry hands (Experiment 2-B). Subjects in both experiments cleaned and dried their hands prior to the experiment and between each trial. For Experiment 2-A the subjects (2 men and one woman) each held the thermal paper for different lengths of time: 2, 15, 30, 45, 60 or 240 sec (in 6 separate trials for each subject). Both hands were wetted by applying three “squirts” of Purell to each hand, and the hands were then briefly rubbed together to distribute the hand sanitizer evenly across both palms and fingers, but the sanitizer was not allowed to dry prior to holding the receipt paper. In experiment 2-B the subjects (2 men and 2 women) held the receipt with dry hands for 60 or 240 sec (2 separate trials for each subject). In both experiments an 8×12 cm portion of thermal paper cut from an unused receipt roll that was obtained from a local merchant (previously identified as containing 27.2 mg BPA/g paper) was placed BPA-coated (print surface) side down into the right hand. The hand was swiped 3 times each with 3 ethanol-soaked Kimwipes, and BPA was extracted from the Kimwipes with methanol and measured by HPLC with CoulArray detection.

### Experiment 3: Serum and urine BPA in men and women before and after transdermal and oral exposure to BPA from thermal receipt paper after using hand sanitizer

**Figure 2:**
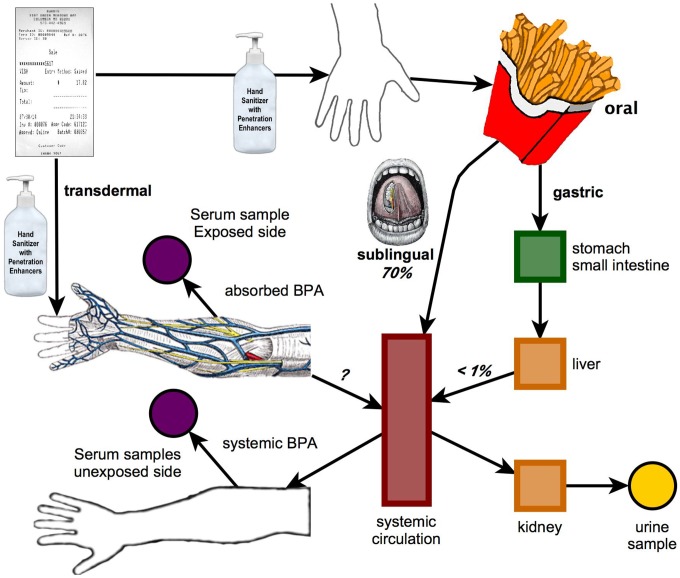
Schematic diagram of the protocol for Experiment 3 in which thermal receipt paper containing BPA was held with a hand wet from using Purell hand sanitizer, after which the subjects picked up 10 French fries and ate them, resulting in both oral and transdermal routes of exposure. Of the 5 male and 5 female subjects, 7 subjects had serum collected from the cubital vein in the arm with a contaminated hand that contained the BPA from holding thermal paper. Three subjects had blood collected from the cubital vein in the unexposed arm that did not have BPA on the hand throughout the 90-min test period during which blood was collected. Urine samples were obtained before and at the end of the test period.

The objective of this experiment was to measure the transfer of BPA from thermal paper receipts to hands, and the amount of BPA remaining on the surface of a hand 90-min later, after using Purell hand sanitizer (as described in the prior experiment) in 5 male and 5 female subjects. In addition, we measured the amount of BPA transferred from a BPA-contaminated hand to 10 French fries, and measured blood and urine concentrations of uBPA, BPA-G and BPA-S before and after ingestion of the French fries and BPA absorption through skin. The design of the study is shown in Figure 2.

The background level of BPA on the dominant hand was determined when the subjects first arrived at the CRC. The dominant hand was swiped 3 times with 3 separate Kimwipes soaked with ethanol, from which we extracted BPA for analysis by HPLC with CoulArray detection, and the hands were then cleaned. The subjects’ weight and height were determined, after which they provided a baseline urine specimen, an IV port was inserted into the cubital vein, and a baseline blood sample was collected. Purell hand sanitizer was applied to the hands as described in Experiment 2. An 8×12 cm piece of thermal paper cut from an unused receipt roll (used in Experiment 2) was then placed BPA-coated side down into each hand with the hands still wet. The subjects held the receipt papers for 4 min in each hand. The dominant arm of each subject was determined based on whether the person was right or left handed, and in this experiment the non-dominant hand remained contaminated with BPA for the duration of the experiment. Blood was collected from the cubital vein in the contaminated arm of one set of subjects (N = 7) and from the cubital vein in the non-contaminated arm of other subjects (N = 3). We note that the phlebotomist did not handle the thermal paper for either Experiment 3 or Experiment 4. The study coordinator who did handle the paper wore gloves to do so and did not touch the blood tubes of other equipment. A separate person swiped the subjects’ hands after thermal paper exposure and wore fresh gloves for each swipe session and discarded them immediately afterwards.

French fries that had been purchased from a local fast food restaurant and had been found to not contain detectable BPA were briefly warmed in a toaster oven. Immediately after holding the thermal receipts in each hand, the subjects picked up a French fry in each hand, and held both fries for 10 sec. The fry held in the dominant hand was placed into a labeled glass tube, and the fry that was held in the non-dominant hand was eaten. A total of 10 French fries was handled by each hand and either placed in a test tube or eaten using this same procedure. Approximately 4 min elapsed between removal of the receipt paper from the hand and consumption of the last French fry. Thus, it took about 8 min from the time that the thermal receipt paper was first touched and consumption of the last French fry.

After the last French fry was consumed, the subject’s dominant hand was swiped with 3 ethanol-soaked Kimwipes to clean BPA off the hand and for determination (by extracting BPA from the Kimwipes) of the amount of BPA remaining on the hand immediately after holding the 10 French fries that were placed into test tubes. The non-dominant hand was not cleaned after holding the receipt paper and eating French fries, and thus was a continuing source of transdermal BPA exposure over the following 90-min period of blood collection.

Blood samples were collected from the cubital vein from the still contaminated arm of 7 subjects, 4 males and 3 females, and from the uncontaminated arm of 3 subjects, one male and 2 females. The blood collected from the BPA-contaminated arm provided direct information about BPA absorbed from the hand on which BPA remained for 90 min, since the cubital vein is one of the major veins draining the hand; this blood is not subject to first-pass liver metabolism prior to going to the heart and being transported in the arterial circulation to tissues. The blood collected from the uncontaminated arm provided information about BPA in the systemic (mixed) circulation.

Blood was collected from the IV port before holding the thermal paper (baseline) and at 15, 30, 60 and 90 min after consumption of the last French fry. The non-dominant contaminated hand (from which the French fries were eaten) was not allowed to touch anything during the 90-min after holding the receipt paper and then picking up the 10 French fries; this hand was swiped with 3 ethanol-soaked Kimwipes after the final 90-min blood collection at the end of the study. After these swipes were obtained, both hands were thoroughly cleaned and a second urine sample was collected.

### Experiment 4: Serum and urine BPA in men and women before and after transdermal exposure to BPA from thermal receipt paper with dry hands

The objective of this study was to examine the amount of BPA transferred to a clean dry hand and then present in serum and urine without using hand sanitizer. In this study we examined 12 adult men and 12 adult women subjects. The subjects washed and dried their hands and provided a baseline blood and urine sample as described in Experiment 3. The non-dominant hand was swiped 3 times each with 3 ethanol-soaked Kimwipes to obtain a baseline measure of BPA on the hand prior to holding a thermal receipt. After the hand was dry, subjects held an 8×12 cm piece of thermal receipt paper (from the roll used in Experiment 1) with the non-dominant dry hand for 4 min. Thirty minutes later a second blood sample was collected from the contaminated arm, after which the BPA was swiped from the contaminated hand with ethanol-soaked Kimwipes as described previously. As above, the contaminated hand was not allowed to touch anything during the 30-min period prior to the second blood collection. The hands were washed, and a second urine sample was collected 60 min after holding the receipt paper.

## Results

### Experiment 1: Measurement of BPA and BPS in thermal receipt paper

Thermal receipts were collected at stores, bars and restaurants in mid-Missouri. Of the 50 receipts, 22 (44%) contained high levels of BPA (Table 1). High levels of the BPA replacement chemical BPS were found in 26 (52%) of the receipts, and 2 receipts contained an unidentified chemical as the print developer [18]; see Section S2 in File S1 for individual values. These findings suggest that BPS is now as commonly used as BPA as a developer in thermal receipt paper. Note that these receipts had been obtained with purchases, while the receipt paper used in Experiments 2, 3 and 4 came from an unused roll of thermal paper that we determine contained BPA as the developer.

**Table 1:** BPA and BPS concentrations in 50 thermal paper receipt samples.

Chemical in paper	mg/g receipt	mg/8×12 cm receipt
BPA-positive (44%)	19.6±1.0	9.0±0.4
	(11.5–26.3)	(6.1–11.3)
BPS-positive (52%)	23.5±0.7	10.8±0.3
	(15.2–30.1)	(7.1–13.2)

Two (4%) of 50 papers tested did not contain either BPA or BPS and did not show any estrogenic activity in a MCF-7 breast cancer cell proliferation assay (data not shown). Values are mean±SEM, with the range of measured values given in parentheses. See Section S2 in File S1 for individual receipt data.

### Experiment 2: BPA transferred to a hand with and without using hand sanitizer due to holding a thermal receipt for different lengths of time

**Figure 3:**
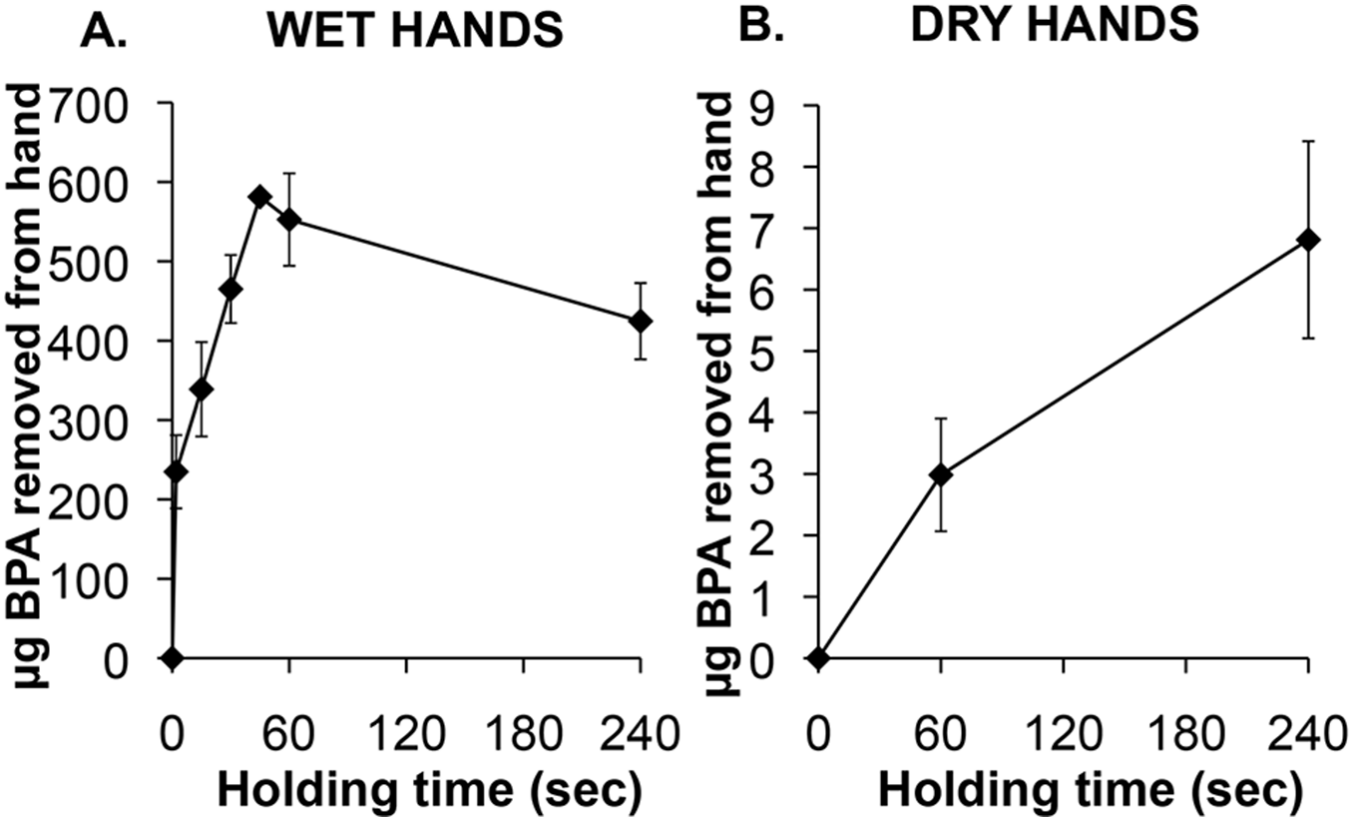
Effect of length of time holding an 8×12 cm thermal receipt on the amount of BPA (µg) swiped from the hand, when hands were pre-wetted with hand sanitizer (Panel A) or left dry (Panel B). The hand was swiped with KimWipes wetted with ethanol to remove the BPA from the surface of the palm and fingers.

The data shown in Figure 3-A reveal that after using Purell hand sanitizer with the hand still wet, the maximum amount of BPA swiped from the palm and fingers of the hand (581 µg BPA) occurred after holding a receipt for 45 sec. After holding a receipt for 2 sec, 40% (235 µg BPA) of maximum was recovered from the hand, and within 15 sec 58% (339 µg BPA) of maximum was recovered from the hand. The decrease in BPA swiped from the hand between 45 sec and 4 min to 73% of maximum (425 µg BPA) may have been due to absorption into skin occurring at a greater rate than transfer to the skin from the thermal receipt.

The data in Figure 3-B show that holding a thermal receipt with dry hands resulted in dramatically lower amounts of BPA being extracted from the receipt relative to the amounts extracted immediately after using hand sanitizer. The ratio of the extracted BPA swiped from the wet vs. dry hand was higher at 60 sec (ratio = 185) than at 240 sec (ratio = 51), reflecting the fact that while the amount of BPA swiped from a wet hand decreased between 60 and 240 sec, the levels increased over this time when the hand was dry, likely due to a reduced rate of absorption with dry relative to wet hands.

### Experiment 3: Serum and urine BPA before and after transdermal and oral exposure to BPA from thermal receipt paper after using hand sanitizer

We measured the amount of BPA swiped from the dominant hand after using hand sanitizer, holding a receipt and then eating 10 French fries, which took 8 min. BPA levels were not significantly different for the 5 males (mean±SEM: 126±19 µg) and the 5 females (mean±SEM: 128±10 µg). These levels measured at about 8-min after first touching the thermal receipt paper were lower than levels measured at 45 sec and 4 min in Experiment 2 (Figure 3-A), which likely reflects rapid transdermal absorption of BPA due to the use of hand sanitizer as well as some of the BPA having been transferred to the French fries. Importantly, females transferred significantly more (58±19 µg) BPA from their dominant hand to the 10 French fries than males (15±3 µg; Mann-Whitney U; P<0.05), resulting in females having a significantly higher oral BPA dose than males between 4–8 min after applying the hand sanitizer.

**Figure 4:**
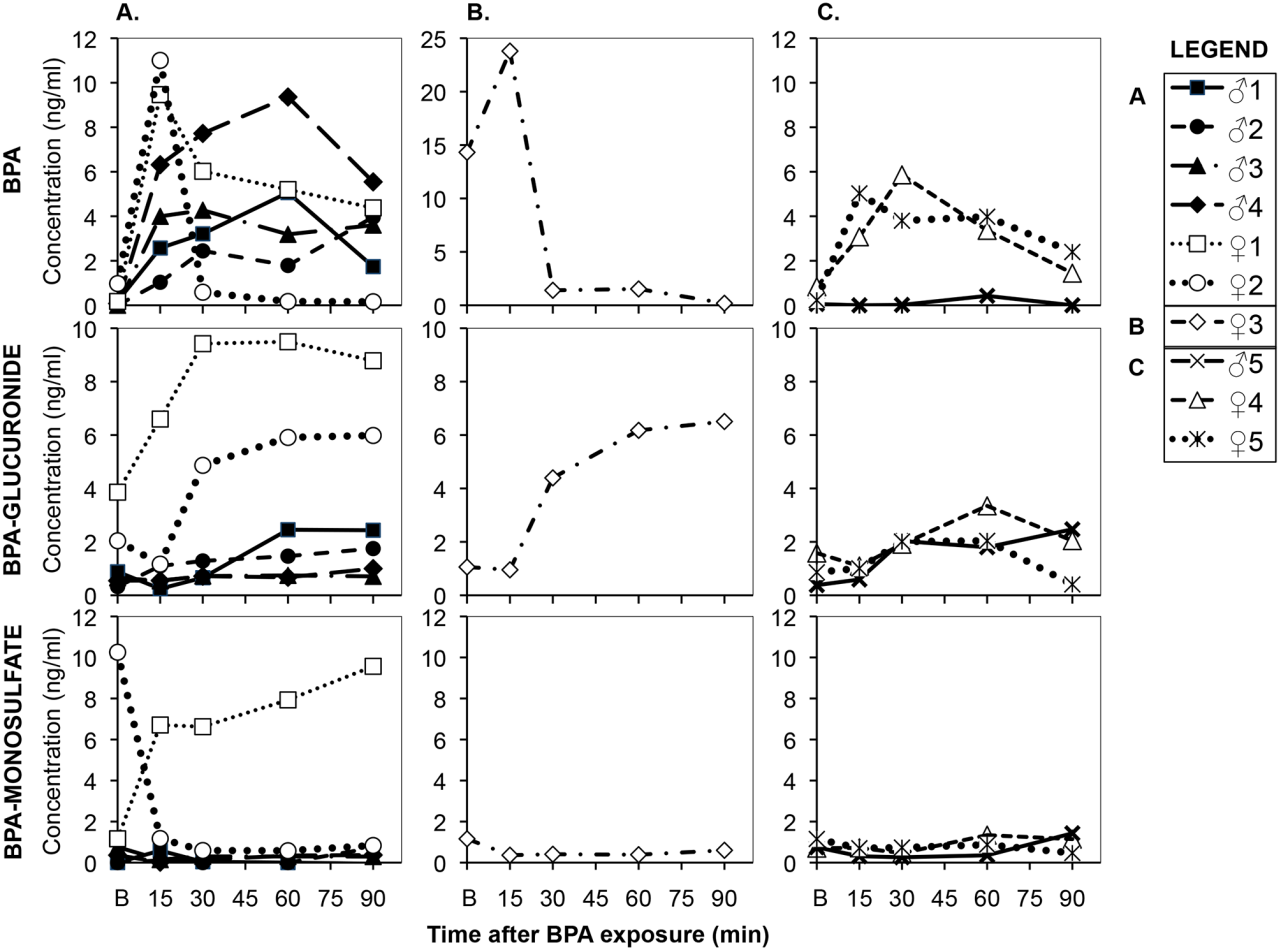
Individual serum profiles of BPA, BPA-G and BPA-MS in men and women prior to (B  = baseline levels) and after holding BPA-containing receipt paper for 4 min followed by picking up and eating 10 French fries over about 4 min with a BPA-contaminated hand. The BPA then remained on the contaminated hand throughout the following 90-min period of blood collection (blood was collected between 15–90 min after eating the last French fry). Panel A: data for serum BPA collected from the contaminated arm with BPA remaining on the hand for 4 males and 2 females that had very low baseline serum uBPA. Panel B: serum BPA data collected from the contaminated arm from Female #3 who had a high baseline serum concentration of uBPA. Panel C: serum BPA data for one male and 2 females who had systemic blood collected from the uncontaminated arm.

Since the participants had been instructed to avoid known sources of BPA, such as canned products, and instructed not to touch thermal paper, 9 of the 10 subjects had undetectable BPA on their dominant hand prior to washing their hands when they first arrived at the Clinical Research Center; none of the subjects was a cashier. However, one female had 0.9 µg of BPA extracted from her hand upon arriving at the CRC, and she was also found to have a very high background concentration of serum uBPA (14.3 ng/mL) prior to holding the thermal receipt paper (subject #3; Figure 4-B). This was the only female subject who was menstruating and thus using products to control menstrual flow, and she also indicated use of hand and body lotion 7–9 times in the prior 48 hr, which was more than any other female or male subject (see Section S3 in File S1). However, even though female #3 (Figure 4-B) had very high background serum uBPA, she showed a dramatic 9.5 ng/mL increase relative to baseline in serum uBPA after holding the thermal receipt and eating 10 contaminated fries at the 15 min blood collection time (15 min after consuming the last French fry). The increase relative to baseline in serum uBPA for female #3 was thus virtually identical to the maximum increase (relative to baseline) found for the other 2 females who had low baseline serum uBPA levels and that were tested in the same way (blood was collected from the BPA contaminated arm; Figure 4-A; Table 1).

### Experiment 3-A: Collection of blood from the cubital vein in the contaminated arm with BPA remaining on the hand throughout the 90-min test period

The data for female subject #3 are not included in the pharmacokinetic data (Table 2) calculated for the remaining 6 subjects that had blood collected from their contaminated arm but who had undetectable baseline levels of BPA on their hands when they first entered the CRC and also had very low baseline uBPA in serum (0.23±0.15 ng/mL; N = 6]. These 6 subjects showed a dramatic increase in serum uBPA after holding the thermal receipt and eating 10 contaminated fries. Females had a greater Cmax and maximum increase relative to baseline in serum uBPA and BPA-G than males after holding the thermal paper, while males reached peak levels of uBPA (Tmax) later than females. The average uBPA value, based on the area under the concentration-time curve [AUC (0–90 min)] did not differ between males and females, while for BPA-G, the AUC (0–90 min) was greater for females than males. The ratio of BPA-G/uBPA based on the average AUC (0–90 min) was very low (0.35±0.12 for males and 1.82±0.30 for females), consistent with routes of absorption of BPA (dermal and sublingual) that bypass first pass metabolism [19], [20].

**Table 2:** Unconjugated BPA (uBPA) and glucuronidated BPA (BPA-G) pharmacokinetic parameters for 4 male and 2 female subjects who held thermal receipt paper and ate French fries after using hand sanitizer (shown in Figure 5-A).

Analyte	Parameter	Male	Female	All
Serum uBPA	Baseline (ng/ml)	0.06±0.04	0.57±0.28	0.23±0.15
	Cmax (ng/ml)	5.66±1.25	10.24±0.77	7.19±1.26
	Maximum increase (ng/ml)	5.60±1.24	9.66±0.37	6.95±1.17
	Range of increase (ng/ml)	3.96–9.28	9.29–10.03	3.96–10.03
	Tmax (min)	60.00±12.25	15.00±0.00	45.00±12.25
	Range of Tmax	30.00–90.00	15.00	15.00–90.00
	Average AUC (0–90 min) (ng/ml)	3.92±1.08	3.85±0.04	3.90±0.75
Serum BPA-G	Baseline (ng/ml)	0.60±0.10	2.95±0.91	1.38±0.55
	Cmax (ng/ml)	1.49±0.39	7.74±1.75	3.57±1.42
	Maximum increase (ng/ml)	0.89±0.36	4.79±0.85	2.19±0.88
	Range of increase (ng/ml)	0.09–1.58	3.94–5.63	0.09–5.63
	Tmax (min)	75.00±8.66	75.00±15.00	75.00±6.71
	Range of Tmax	60.00–90.00	60.00–90.00	60.00–90.00
	Average AUC (0–90 min) (ng/ml)	1.05±0.21	6.48±1.93	2.86±1.25
	BPA-G/uBPA AUC (0–90 min) (ng/mL)	0.35±0.12	1.82±0.30	0.84±0.33
Urine Total BPA	Baseline (ng/ml)	0.15±0.04	1.10±0.58	0.46±0.24
	Baseline (µg/g creatinine)	0.20±0.09	1.22±0.24	0.54±0.19
	90 min (ng/ml)	23.36±6.66	10.62±3.16	19.11±4.32
	90 min (µg/g creatinine)	18.20±5.33	40.93±22.56	25.77±8.56

For these subjects blood was collected from the arm draining the hand that remained contaminated with BPA throughout the 90-min period of blood collection. Urine total BPA at baseline and at 90 min are presented as both actual concentration (ng/ml) and creatinine adjusted (µg BPA/g creatinine) values.

**Figure 5:**
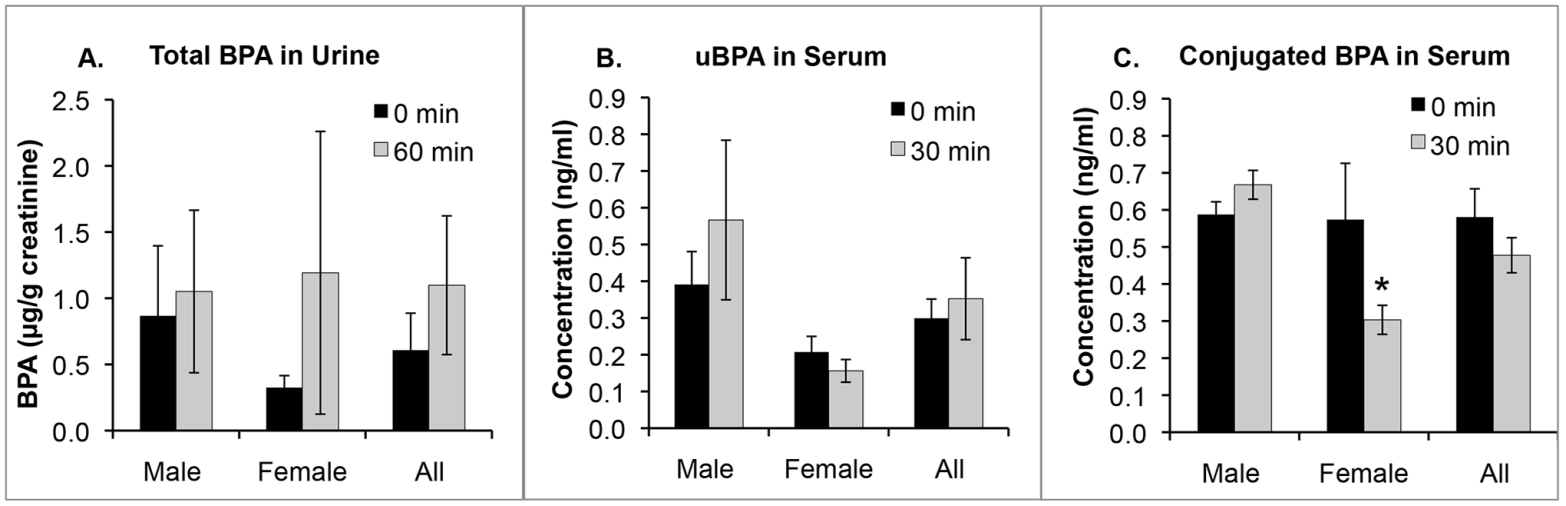
BPA in urine and serum of 12 men and 12 women who held thermal receipt paper with dry hands for 4 min, Panel A: the total concentration of BPA in urine (expressed relative to creatinine) at baseline and 60-min after holding the thermal receipt. Panel B: unconjugated BPA (uBPA) in serum at baseline and 30 min after holding the thermal receipt. Panel C: conjugated BPA (BPA-G and BPA-S) in serum at baseline and 30 min after holding the thermal receipt paper. * = significant difference between males and females (P<0.001).

For the one female (Female #3; Figure 5-B) who had very high baseline serum uBPA (data not included in this table), the average serum AUC (0–90) values for uBPA and BPA-G were 6.05 and 4.49 ng/ml, respectively, and urine total BPA levels at baseline and at 90 min were 0.41 and 41.41-µg/g creatinine, respectively.

Only female #1 (Figure 4-A) showed a marked increase in serum BPA-S relative to baseline, revealing that while BPA-G is the major conjugated metabolite of BPA in most men and non-pregnant women, some individuals do form significant amounts of BPA-S. Urine total BPA (unconjugated and conjugated) increased dramatically between baseline and 90 min after handling thermal paper, although unlike the serum data, there was no difference between males and females (Table 2).

### Experiment 3-B: Collection of blood from the cubital vein in the uncontaminated arm to measure BPA in mixed systemic blood throughout the 90-min test period

We also obtained data from 3 subjects who had the same procedures described above except that they had blood collected from the cubital vein in the opposite uncontaminated arm that did not have BPA remaining on the hand during the 90-min period of blood collection. The 3 subjects consisted of one male and 2 females (age 22.3±0.9 yrs, BMI 26.0±0.9). While the baseline serum uBPA levels were very low (Figure 4-C; Table 3), the average serum uBPA AUC (0–90 min) for the two female subjects (Table 3) was similar to the data from the other 7 subjects discussed in Experiment 3-A (Table 2). Even for the male subject with low serum uBPA after holding thermal receipt paper, BPA-G in serum increased between baseline and 90 min (Figure 4-C), and urine total BPA increased dramatically over the 90-min test, similar to the increase in total urine BPA in the women (Table 3). These findings show that high levels of uBPA could be detected in the systemic circulation of subjects after holding thermal receipt paper and eating 10 French fries. Levels of total BPA in urine at baseline or 90 min in these 3 subjects (Table 3) were similar to levels measured in the other 7 subjects (Table 2).

**Table 3:** Unconjugated BPA (uBPA) and glucuronidated BPA (BPA-G) pharmacokinetic parameters for one male and two female subjects who held thermal receipt paper and ate French fries after using hand sanitizer (shown in Figure 5-C).

Analyte	Parameter	Male	Female	All
Serum uBPA	Baseline (ng/ml)	0.06	0.53±0.30	0.37±0.24
	Cmax (ng/ml)	0.42	5.44±0.42	3.77±1.69
	Maximum increase (ng/ml)	0.37	4.91±0.13	3.40±1.53
	Range of increase (ng/ml)	0.37	4.80–5.03	0.37–5.03
	Tmax (min)	60.00	22.50±7.50	35.00±13.23
	Range of Tmax	60.00	15.00–30.00	15.00–60.00
	Average AUC (0–90 min) (ng/ml)	0.15	3.47±0.06	2.36±1.11
Serum BPA-G	Baseline (ng/ml)	0.38	1.22±0.36	0.94±0.35
	Cmax (ng/ml)	2.47	2.69±0.65	2.62±0.39
	Maximum increase (ng/ml)	2.09	1.47±0.30	1.68±0.27
	Range of increase (ng/ml)	2.09	1.18–1.77	1.18–2.09
	Tmax (min)	90.00	60.00	70.00±10.00
	Range of Tmax	90.00	60.00	60.00–90.00
	Average AUC (0–90 min) (ng/ml)	1.65	1.87±0.38	1.80±0.23
	BPA-G/uBPA AUC (0–90 min) (ng/mL)	11.00	0.54±0.12	4.03±3.49
Urine Total BPA	Baseline (ng/ml)	1.92	0.38±0.15	0.89±0.52
	Baseline (µg/g creatinine)	1.17	0.21±0.08	0.53±0.40
	90 min (ng/ml)	27.86	29.34±0.35	28.85±0.54
	90 min (µg/g creatinine)	27.35	14.11±1.16	18.53±4.46

For these subjects mixed systemic blood was collected from the uncontaminated arm over the 90 min after BPA exposure. Urine total BPA at baseline and at 90 min are presented as both actual concentration (ng/ml) and creatinine adjusted (µg/g creatinine) values.

### Experiment 4: Serum and urine BPA in men and women before and after transdermal exposure to BPA from thermal receipt paper with dry hands

We conducted this study with 12 male (age 27.7±1.6 yrs, BMI 26.9±0.9) and 12 female (age 25.8±1.6 yrs, BMI 25.2±1.5) subjects. Male and females subjects held a single 8×12 cm piece of thermal receipt paper in the non-dominant hand for 4 min, but unlike the prior experiment, no hand sanitizer was used prior to holding the thermal paper with a dry hand. No BPA was detected on the hands of any subject after washing and drying the hands prior to holding the thermal receipt paper. We did not determine the amount of BPA transferred to the hand immediately after holding the thermal receipt paper, since we had previously examined this (Figure 3-B). However, 30 min after holding the thermal paper BPA was swiped from the surface of the hand: for men (5.5±1.7 µg; range: 0.8–22.5 µg) and women (6.1±0.8 µg; range: 1.3–10.6 µg). There was no difference between males and females in the urine total BPA concentration at baseline or at 60 min, and there was also no difference between total urine BPA at baseline vs. 60 min for either males or females (Figure 5-A). There was a tendency (based on 2-tailed t-tests) for males to have higher serum uBPA at baseline (P = 0.08) and after 30 min (P = 0.06) relative to females (Figure 5-B). While there was no sex difference in conjugated BPA (cBPA), consisting of both BPA-G and BPA-S, at the baseline blood collection (Figure 5-C), at 30 min after holding the thermal receipt males had significantly higher serum conjugated BPA than females (ANOVA; P<0.001).

## Discussion

Our data provide the first evidence that the use of very large amounts of free BPA as a developer on the print surface of thermal paper (∼20 mg BPA/g paper) could be an important factor in accounting for the high levels of bioactive serum uBPA and urine total excreted BPA reported previously in various human populations [21]. We conducted this study to mimic aspects of the behavior of people in a fast-food restaurant where we have observed people using hand sanitizer and handling a thermal receipt for variable periods of time prior to picking up and eating food with their hands. In Figure 3 we show that holding a receipt for 45 sec immediately after using hand sanitizer containing dermal penetration enhancing chemicals resulted in the maximum amount of BPA that was swiped from the palm and fingers (581 µg BPA). After holding the receipt for 2 sec 40% of maximum was recovered from the hand, and within 15 sec 58% of maximum was recovered. Between 45 sec and 4 min, the amount of BPA recovered from the surface of the hand decreased, which may have been due to absorption into skin occurring at a greater rate than transfer to the skin from the thermal receipt. These findings show that a very large amount of BPA is transferred from thermal paper to a hand as a result of holding a thermal receipt for only a few seconds immediately after using a product with dermal penetration enhancing chemicals. The data in Figure 3-A also suggest that transdermal BPA absorption is very rapid due to the penetration enhancing chemicals in the hand sanitizer that we used, and thus measurement of BPA swiped from the surface of the hand likely underestimates the actual amount of free BPA transferred from the print surface of thermal paper. We note that since the thermal receipt paper is sold in rolls, the non-print surface has BPA transferred to it from the print surface (Figure 1). By swiping the two surfaces with ethanol on Kimwipes, the print surface was found to contain an 8.7-fold greater amount of BPA relative to the non-print surface of the thermal receipt paper roll used in these experiments (data not shown).

In both men and women there was a dramatic increase in serum uBPA after using hand sanitizer with dermal penetration enhancing chemicals and then holding thermal receipt paper and eating French fries with the BPA-contaminated hand (Figure 4-A and 4-B). While the sample size was small, our data suggest higher maximum serum levels (Cmax) for females and a greater maximum increase relative to baseline for both uBPA and BPA-G (the primary conjugated BPA metabolite) for females relative to males (Table 2). This finding was related to a greater transfer of BPA from the hand to the French fries and thus a greater oral dose (by about 4 fold) in females relative to males. However, we cannot rule out that the skin of females also allows greater transdermal transport of BPA relative to males due to sex differences in skin permeability [13]. In fact, our data are consistent with the hypothesis that a combination of both transdermal and buccal/sublingual absorption (Figure 2) resulted in the dramatic increase in both serum uBPA (Figure 4-A and 4-B) and total BPA excreted in urine (Table 2). The profile of serum uBPA suggests that females absorbed BPA more rapidly than males (Figure 4-A), consistent with females having a shorter time to reach the maximum serum level (Tmax) of uBPA (Table 2). The later Tmax in men than in women is consistent with men having a thicker stratum corneum (the outermost layer of the epidermis) relative to women [22], [23]. In addition to skin thickness, another possible explanation for the sex differences we observed (Table 2) would be a greater use of skin moisturizers in females than in males, which could impact both the transfer of BPA to the hand from the surface of thermal paper as well as transdermal absorption of BPA. Our finding that males tended to have higher serum uBPA and had significantly higher serum conjugated BPA than women at 30 min after holding a receipt with a dry hand (Figure 5-B and 5-C) requires further study, since the more rapid absorption of BPA found for women after using hand sanitizer (Figure 4-A and 4-B) would have been missed at the 30-min blood collection time.

For the 3 females that had blood collected from the cubital vein in the same arm with the BPA contaminated hand (Figure 4-A and 4-B; Table 2), the maximum increase in serum uBPA relative to baseline (∼10 ng/mL) was about two-times greater than the maximum increase found in the other two females whose blood was collected from the opposite uncontaminated arm (∼5 ng/mL; Table 3; Figure 4-C). This difference between blood collected from a vein draining the contaminated hand vs. blood from the opposite uncontaminated arm (reflecting uBPA in the systemic circulation) suggests that a substantial amount of uBPA in blood from the contaminated arm was due to the BPA that was transdermally absorbed before its mixing in the general circulation. Our data from the cubital vein draining the contaminated hand that had BPA remaining on it for 90 min thus support the hypothesis of a higher arterial than mixed venous blood BPA concentration during the dermal absorption phase in the framework of a physiologically based pharmacokinetic (PBPK) model [24]. The hypothesis that served as the basis for collecting blood from the same arm in which BPA was being absorbed is that a portion of the BPA contaminated blood would be transported from the contaminated hand through the cubital vein and then to the heart. Subsequently, the contaminated blood would enter the arterial circulation and be transported to the tissues in the body, including endocrine target tissues. This leads to the prediction that during the dermal absorption of BPA, the BPA concentration in arterial blood is likely more relevant to consider in terms of exposure than the BPA concentration in the mixed venous blood, because blood in a vein draining the contaminated hand is not subjected to clearance by enzymes in the liver prior to reaching endocrine target tissues in arterial blood.

When examining all of our data for serum unconjugated and conjugated BPA, we show significant inter-subject variability in the absorption and clearance of BPA (Figure 4), which was also previously found for the estrogenic drug used in oral contraceptives, ethinylestradiol [25]. A particular concern is that there are individuals who have limited capacity to excrete BPA or other estrogenic compounds; one population at risk is patients with early-stage or advanced kidney disease [26], [27].

The default method of administration of chemicals to animals by regulatory agencies is by intra-gastric gavage, regardless of how the chemical is used or whether there are known non-oral routes of exposure [28]. It is thus not surprising that US and European regulatory agencies [29], [30] have modeled human exposure to BPA based on results from intra-gastric gavage administration of BPA to animals, which results in direct transport of BPA to the liver via the mesenteric vessels and extensive first-pass metabolism (detoxification) in the liver (Figure 2); the result is less than 1% of the gavage administered dose being bioavailable in blood [19], [31]. However, Gayrard et al. [19] found high absorption and bioavailability (∼70%) of BPA following sublingual administration that was dramatically different than the much lower bioavailability (<1%) of BPA following gavage administration in a parallel experiment. These findings directly challenge predictions that it is not possible to find the high blood levels of biologically active uBPA that have actually been measured in numerous human biomonitoring studies [21], [32] but are currently being rejected for use in risk assessments by the US-Food and Drug Administration (US-FDA) as not plausible [31].

In contrast to the extremely high ratio of BPA-G to uBPA (>100∶1) predicted by gavage exposure studies due to rapid phase 2 metabolism in the liver, the average ratio of BPA-G to uBPA in our Experiment 3 was 0.84±0.33 based on the average AUC (0–90 min) for the 6 subjects with blood collected from the arm with the contaminated hand (Figure 5-A; Table 2); this ratio was also low for the subjects with systemic blood collected from the uncontaminated arm (Table 3). These findings indicate that the primary route of BPA exposure was not via gastrointestinal absorption after eating the BPA-contaminated French fries, since this ratio would be predicted to exceed 100∶1 [31], [33]. One reason may have been that, in addition to transdermal absorption, the BPA transferred to the French fries would have been on the surface of the fries and thus easily absorbed by the highly vascularized epithelium in the mouth [19].

In the present study we measured total BPA in urine to be able to relate our findings to a very large epidemiological literature showing BPA in urine to be correlated with abnormal development and diseases in children and adults [5], [34]. The geometric mean for adults in the 95th percentile for total BPA in urine reported in NHANES 2003/4 was about 11 µg/g creatinine [2] and the range of values at the 95^th^ percentile include values we measured here. Periodically, BPA levels exceeding those we found are reported in studies that measured BPA in urine [21], and it is possible that those assays are of people who had very recently been exposed to BPA in a manner similar to our experiment.

Our findings thus provide evidence regarding how some people could be found to have very high urine levels of BPA. Importantly, the amount of total BPA in urine was ∼20 µg BPA/g creatinine (∼20 ng/mL urine uncorrected for creatinine). This high level of urine total BPA collected 90 min after using hand sanitizer and holding a thermal receipt (Table 2 and Table 3) has been associated with a significant increase in the likelihood of developing cardiovascular disease and type 2 diabetes [35], [36]. BPA levels in human urine have also been related to a wide range of other diseases in over 60 human epidemiological studies [5], [34]. Published findings include: reproductive effects in women (polycystic ovary syndrome, altered ovarian response to hormones, reduced fertilization success, implantation failure, endometrial disorders, reduced embryo quality, miscarriage, premature delivery and breast cancer), reproductive effects in men (reduced libido, sperm quality, altered sex hormone concentrations and embryo quality), altered thyroid hormone concentrations, obesity, impaired liver function, impaired immune and kidney function, inflammation, and neurobehavioral deficits such as aggressiveness, hyperactivity and impaired learning [5], [34]. The estimate of the costs per year of additional cases of just cardiovascular disease in the USA attributable to BPA is 1.5 billion dollars [37].

In a study that involved handling thermal receipts (without using hand sanitizer) continuously for 2 hr, which would be relevant for a cashier, there was a significant increase in urine total BPA relative to baseline [38]. This finding is consistent with prior data that cashiers have higher levels of BPA in urine than the general public [39]. Blood concentrations of BPA were not determined in these studies, and they also did not take into account that perhaps 50% of the receipts handled may have contained BPS rather than BPA (Table 1). The results of these studies indicate that with repeated handling of thermal receipts in an occupational setting, even without the use of hand sanitizer, there is a significant increase in BPA exposure. Future studies involving handling of thermal paper need to include analysis of the thermal paper to determine if the developer used is BPA or some other chemical. Related to the issue of occupational exposure to BPA is our observation that at least one big-box store in Columbia, Missouri provides hand sanitizer dispensers for use by all cashiers, and our data suggest this can not only markedly increase transfer of BPA from the thermal paper to hands but also increase transdermal absorption of BPA.

Our findings that thermal receipt paper is a potential source of high exposure to BPA are supported by data showing that BPA readily leaches from thermal receipts and thus likely contaminates anything that a receipt contacts. Thus, environmental contamination caused by the use of unpolymerized (free) BPA in thermal paper is widespread [7]. The dermal penetration enhancing chemicals present in personal care products as well as hand sanitizers cause a breakdown of the dermal barrier leading to an increase in transdermal absorption [8], [9]. While BPA was reported to be absorbed through pig and human skin *in vitro* [40], our data show after holding a receipt for 60 sec, there was 185-times more BPA transferred to a wet hand due to holding thermal receipt paper immediately after using hand sanitizer with penetration enhancing chemicals as opposed to when the hands were dry (Figure 3). The specific mixtures of chemicals used in products will impact transdermal exposure to environmental chemicals such as BPA [8], [9], and additional research is needed to determine the degree to which alcohols and other chemicals impact exposures. This is important because when soap and water are not available, hand sanitizers are recommended to reduce infectious disease transmission [http://www.cdc.gov/handwashing/when-how-handwashing.html]. It is also important to determine the length of time after using skin-care products with dermal penetration enhancing chemicals that there is an impact on absorption of environmental contaminants.

The issue of assay performance is obviously very important and was examined using a round robin validation process in Europe for a number of chemicals, including BPA, which identified that some laboratories were able to accurately assay uBPA and other chemicals without contamination, while other laboratories were unable to assay uBPA or other chemicals accurately [41]. Importantly, our findings reported here are based on measurement of uBPA with a sensitive, validated, contamination-free LC/MSMS assay. Specifically, our laboratory is one of three in the U.S. that recently successfully completed a NIH-sponsored round-robin measuring uBPA in human serum [17]. In addition, in the present experiment, the time course of blood uBPA concentrations after a controlled BPA exposure in our university Clinical Research Center (Figure 4) is not consistent with any spurious contamination; this conclusion was also supported by the use of field blanks. It is thus clear that the prediction that any finding of uBPA in human serum must be due to sample contamination [31] is not valid, even though a few investigators report being unable to control BPA contamination in their assays [42], [43]. Supporting our conclusion is a report from the CDC in which sources of contamination were identified and systematically eliminated during the successful development of assays for BPA and three other chemicals [44]. The issue of the potential for assay contamination is thus not unique to BPA and simply requires the use of standard assay procedures and appropriate controls that should be routinely employed.

## Conclusions

Thermal paper requires a chemical in the surface coating as a print developer. The current preferred developers, BPA and BPS, have both been shown to have estrogenic activity [45], [46]. This is leading to widespread exposure to both of these endocrine-disrupting chemicals [7], [47], and BPS is more persistent in the environment relative to BPA and is thus an unacceptable replacement for BPA [18], [48]. A recent EPA report examined 19 alternative chemicals, including BPS, that could potentially replace BPA as a developer in thermal paper and concluded that “No clearly safer alternatives to BPA were identified in this report; most alternatives have Moderate or High hazard designations for human health or aquatic toxicity endpoints” [18]. The report identified that “decision makers may wish to consider alternative printing systems”. Two of the papers screened for our current study employed a developer other than BPA or BPS that was not estrogenic in a MCF-7 human breast cancer cell proliferation assay (data not shown), but lack of estrogenic activity does not imply safety, as indicated in the EPA report.

Thermal paper is a major source of BPA contamination in recycled paper, and its use results in the widespread contamination of other products and the environment [49] due to the presence of large amounts of free, unpolymerized BPA in the surface coating of thermal paper (Figure 1). Further, our findings are consistent with other data reporting that BPA can be transferred from the surface of thermal paper to items it contacts. Because no safe alternatives to the use of BPA or its primary replacement chemical BPS in thermal paper have been identified, our findings provide support for the EPA’s recommendation that thermal paper should be replaced with other safer technologies [18].

Our study provides the first data that thermal paper may be a significant factor in accounting for high levels of bioactive BPA in human serum and total BPA in urine that have been associated with diseases that are increasing in frequency in human populations [21], [34]. Our findings also suggest that the impact of the use of dermal penetration enhancing chemicals in skin care products on transdermal absorption of environmental contaminants should be taken into consideration in risk assessments and should be a priority for future research.

## Supporting Information

File S1 Section S1: Sample handling, extraction and assay methods. Section S2: Table of individual BPA and BPS values for 50 thermal receipt papers. Section S3: List of questions asked each subject.
(DOCX)
