# Supplementary material for: Holding Thermal Receipt Paper and Eating Food after Using Hand Sanitizer Results in High Serum Bioactive and Urine Total Levels of Bisphenol A (BPA)
Source: PLoS One. 2014 Oct 22;9(10):e110509. doi: 10.1371/journal.pone.0110509 (PMC4206219; doi:10.1371/journal.pone.0110509)
Supplement: File S1 — Section S1: Sample handling, extraction and assay methods. Section S2: Table of individual BPA and BPS values for 50 thermal receipt papers. Section S3: List of questions asked each subject. (DOCX) [file pone.0110509.s001.docx]

Holding thermal receipt paper and eating food after using hand sanitizer

results in high serum bioactive and urine total levels of bisphenol A (BPA)

Annette M. Hormann^1^, Frederick S. vom Saal^1^, Susan C. Nagel^2^, Richard W. Stahlhut^1^, Carol L. Moyer^1^, Mark R. Ellersieck^3^, Wade V. Welshons^4^, Pierre-Louis Toutain^5,6^ and Julia A. Taylor^1^

^1^Division of Biological Sciences

^2^Department of Obstetrics, Gynecology and Women’s Health

^3^Department of Statistics

^4^Department of Biomedical Sciences

University of Missouri

Columbia MO 65211, USA

^5^Université de Toulouse

INPT, ENVT, UPS, UMR1331

F- 31062 Toulouse, France

^6^INRA, UMR1331

Toxalim

Research Centre in Food Toxicology

F-31027 Toulouse, France

**S1. PROCEDURAL DETAILS**

**Blood sample collection***.*

Single point blood samples were collected by venipuncture using a vacutainer safety lok needle (Becton Dickinson, Franklin Lakes, NJ; cat # 367292) into uncoated glass vacutainer tubes (Becton Dickinson, cat# 366441). Multiple-point blood samples were collected via IV catheter (Becton Dickinson, cat# 381533) and Carefusion extension tubing (Alaris Products, San Diego, CA) into 10 mL syringes (Becton Dickinson, cat# 309604). Separated serum was transferred to 15 mL centrifuge tubes (Corning Life Sciences, Tewksbury, MA; cat# 430791) for storage at -20^o^C.

**Extraction of BPA and BPA conjugates in serum***.*

Samples were extracted as previously described [[1](#_ENREF_1)] using the method of Coughlin et al. [[2](#_ENREF_2)] with minor modifications. One mL of serum was spiked with an internal recovery standard (C^13^-BPA) and diluted to 6 mL with ammonium acetate (final concentration 200 mM), 20 µl formic acid and HPLC-grade H2O. The diluted sample was passed through a Thermo Hypersep C18 cartridge (Thermo Scientific), previously conditioned with 15 mL methanol and 3 mL H_2_O. The cartridges were washed with 2 mL of 25 mM ammonium acetate and 3 mL H_2_O and dried briefly. Analytes were eluted with 4 mL MeOH. The eluates were dried under N_2_ and reconstituted in 50:50 MeOH:20 mM ammonium acetate for analysis by LC/MSMS.

**Extraction of BPA and BPA conjugates in urine.**

Samples were first incubated in 100 mM ammonium acetate buffer (pH 5.0) overnight, with 100 units b-glucuronidase (Type H-1, Sigma; this enzyme preparation also contains sulfatase activity). The samples were passed through C18 SPE cartridges using the same procedure described for serum (see Supplemental Materials Section S1). The methanol eluate was dried and reconstituted in chloroform:isopropanol (2:1) and then passed through an NH2 SPE cartridge. The extract was dried under nitrogen and reconstituted in 50:50 MeOH:20 mM ammonium acetate for HPLC. The total BPA concentration (representing a combined measure of deconjugated BPA conjugates and free BPA) was measured by LC/MSMS.

**LC/MSMS methods***.*

BPA, BPA-G, BPA-MS and BPS in serum and BPA in urine (after hydrolysis) were quantified by liquid chromatography with mass spectrometry (LCMS/MS) using a Thermo TSQ Quantum Access Max (Thermo Fisher Scientific, Waltham, MA) connected to an integrated Thermo-Accela LC system, as described elsewhere [[1](#_ENREF_1)]. Analytes were detected using electrospray ionization with negative polarity, and conditions (tube lens setting, collision energy) were optimized for each analyte using the instrument software. Separations were performed on a 100x4.6 mm 3 micron Hyperclone HPLC column (Phenomenex, Torrance, CA), at a flow rate of 350 µL/min. A gradient mobile phase was employed using 10:90 acetonitrile and acetonitrile, containing 0.01% ammonia. Thermo LCQuan software was used to autotune, acquire, and process the LC/MS data. BPA, C^13^-BPA, BPA-G, BPA-MS and BPS were detected using selected reaction monitoring for m/z 227>212, m/z 239>224, m/z 403>227, m/z 306>212 and m/z 248>108 respectively, and quantitation was made against standard curves of the analytes at concentrations ranging from 1-200 ng/ml. The limits of quantitation (LOQ) for BPA, BPA-G, BPA-MS and BPS were 0.18, 0.33, 0.11 and 0.37 ng/mL respectively.

**HPLC-CoulArray methods***.*

Methanol extracts of BPA from KimWipes, thermal paper and French fries were diluted in methanol as necessary, and then assayed directly without further sample preparation. Concentrations of BPA in sample extracts were determined by HPLC with an ESA CoulArray 5600 detector. Separation was performed on a reverse-phase 250 mm Prodigy C18 column (Phenomenex), with a mobile phase of 36:24:40 acetonitrile: methanol: 0.05 M sodium acetate buffer (pH 4.8), and with the CoulArray cell potentials set at 325, 400, 720 and 875 mV. Recoveries were determined from parallel-processed blank samples spiked with known amounts of BPA.

**References**

1. Vandenberg LN, Gerona RR, Kannan K, Taylor JA, van Breemen RB, et al. (2014) A round robin approach to the analysis of bisphenol a (BPA) in human blood samples. Environ Health 13: 25.

2. Coughlin JL, Winnik B, Buckley B (2011) Measurement of bisphenol A, bisphenol A ss-D-glucuronide, genistein, and genistein 4'-ss-D-glucuronide via SPE and HPLC-MS/MS. Anal Bioanal Chem 401: 995-1002.

**S2. SUMMARY OF BPA AND BPS VALUES IN EXTRACTED RECEIPTS**

|  |  | BISPHENOL A | | | | BISPHENOL S | | |  |  |
| --- | --- | --- | --- | --- | --- | --- | --- | --- | --- | --- |
|  | Sample ID | ug/cm^2^ | mg/g | mg/3 in^2^ | ug/cm^2^ | | mg/g | mg/3 in^2^ | |  |
| COLUMBIA, MO | 20 | 116.2 | 26.3 | 6.7 | 0.0 | | 0.0 | 0.0 | |  |
| BPA DETECTED | 50 | 118.1 | 25.9 | 6.9 | 0.0 | | 0.0 | 0.0 | |  |
|  | 7 | 112.7 | 24.6 | 6.5 | 0.0 | | 0.0 | 0.0 | |  |
|  | 32 | 111.2 | 24.1 | 6.5 | 0.0 | | 0.0 | 0.0 | |  |
|  | 45 | 111.0 | 23.8 | 6.4 | 0.0 | | 0.0 | 0.0 | |  |
|  | 43 | 99.9 | 21.9 | 5.8 | 0.0 | | 0.0 | 0.0 | |  |
|  | 42 | 93.5 | 21.8 | 5.4 | 0.0 | | 0.0 | 0.0 | |  |
|  | 46 | 99.0 | 21.7 | 5.8 | 0.0 | | 0.0 | 0.0 | |  |
|  | 37 | 98.6 | 20.4 | 5.7 | 0.0 | | 0.0 | 0.0 | |  |
|  | 40 | 88.2 | 20.2 | 5.1 | 0.0 | | 0.0 | 0.0 | |  |
|  | 30 | 86.4 | 19.4 | 5.0 | 0.0 | | 0.0 | 0.0 | |  |
|  | 10 | 86.4 | 18.2 | 5.0 | 0.0 | | 0.0 | 0.0 | |  |
|  | 41 | 77.2 | 16.1 | 4.5 | 0.0 | | 0.0 | 0.0 | |  |
|  | 24 | 93.1 | 15.9 | 5.4 | 0.0 | | 0.0 | 0.0 | |  |
|  | 35 | 81.1 | 14.5 | 4.7 | 0.0 | | 0.0 | 0.0 | |  |
|  | 28 | 63.5 | 11.7 | 3.7 | 0.0 | | 0.0 | 0.0 | |  |
|  | 29 | 64.2 | 11.5 | 3.7 | 0.0 | | 0.0 | 0.0 | |  |
| COLUMBIA, MO | 47 | 0.0 | 0.0 | 0.0 | 131.3 | | 30.1 | 7.6 | |  |
| BPA NOT DETECTED | 51 | 0.0 | 0.0 | 0.0 | 132.0 | | 29.0 | 7.7 | |  |
| DETECTED | 12 | 0.0 | 0.0 | 0.0 | 136.9 | | 27.9 | 7.9 | |  |
|  | 44 | 0.0 | 0.0 | 0.0 | 137.0 | | 27.6 | 8.0 | |  |
|  | 52 | 0.0 | 0.0 | 0.0 | 113.3 | | 26.4 | 6.6 | |  |
|  | 11 | 0.0 | 0.0 | 0.0 | 127.7 | | 26.1 | 7.4 | |  |
|  | 13 | 0.0 | 0.0 | 0.0 | 119.4 | | 25.8 | 6.9 | |  |
|  | 8 | 0.0 | 0.0 | 0.0 | 116.3 | | 25.5 | 6.8 | |  |
|  | 14 | 0.0 | 0.0 | 0.0 | 111.5 | | 25.4 | 6.5 | |  |
|  | 38 | 0.0 | 0.0 | 0.0 | 121.7 | | 25.3 | 7.1 | |  |
|  | 39 | 0.0 | 0.0 | 0.0 | 120.2 | | 25.2 | 7.0 | |  |
|  | 5 | 0.0 | 0.0 | 0.0 | 119.6 | | 24.4 | 6.9 | |  |
|  | 36 | 0.0 | 0.0 | 0.0 | 131.3 | | 24.0 | 7.6 | |  |
|  | 49 | 0.0 | 0.0 | 0.0 | 119.3 | | 22.4 | 6.9 | |  |
|  | 26 | 0.0 | 0.0 | 0.0 | 103.9 | | 21.8 | 6.0 | |  |
|  | 21 | 0.0 | 0.0 | 0.0 | 98.7 | | 21.5 | 5.7 | |  |
|  | 25 | 0.0 | 0.0 | 0.0 | 95.4 | | 21.5 | 5.5 | |  |
|  | 33 | 0.0 | 0.0 | 0.0 | 103.1 | | 21.4 | 6.0 | |  |
|  | 31 | 0.0 | 0.0 | 0.0 | 109.6 | | 21.2 | 6.4 | |  |
|  | 6 | 0.0 | 0.0 | 0.0 | 100.5 | | 20.8 | 5.8 | |  |
|  | 27 | 0.0 | 0.0 | 0.0 | 103.7 | | 20.5 | 6.0 | |  |
|  | 53 | 0.0 | 0.0 | 0.0 | 101.5 | | 19.5 | 5.9 | |  |
|  | 22 | 0.0 | 0.0 | 0.0 | 0.0 | | 0.0 | 0.0 | |  |
|  | 48 | 0.0 | 0.0 | 0.0 | 0.0 | | 0.0 | 0.0 | |  |
| SOUTHERN MO | 1 | 114.8 | 26.2 | 6.7 | 0.0 | | 0.0 | 0.0 | |  |
| BPA DETECTED | 18 | 112.2 | 24.4 | 6.5 | 0.0 | | 0.0 | 0.0 | |  |
|  | 3 | 77.6 | 16.7 | 4.5 | 0.0 | | 0.0 | 0.0 | |  |
|  | 16 | 76.7 | 13.6 | 4.5 | 0.0 | | 0.0 | 0.0 | |  |
|  | 4 | 69.8 | 12.8 | 4.1 | 0.0 | | 0.0 | 0.0 | |  |
| SOUTHERN MO | 19 | 0.0 | 0.0 | 0.0 | 101.0 | | 20.9 | 5.9 | |  |
| BPA NOT DETECTED | 15 | 0.0 | 0.0 | 0.0 | 97.6 | | 21.6 | 5.7 | |  |
| DETECTED | 17 | 0.0 | 0.0 | 0.0 | 92.3 | | 19.3 | 5.4 | |  |
|  | 2 | 0.0 | 0.0 | 0.0 | 73.5 | | 15.2 | 4.3 | | |

**S3: QUESTIONS ANSWERED BY EACH SUBJECT**

**How many times in the past 48 hours did you:**

Answer: 0, 1-3, 4-6, 7-9, 10+

A Eat vegetables from a can?

B Drink from a canned beverage?

C Drink from a reusable hard plastic sport water bottle?

D Drink a soda purchased from a convenience store in a plastic cup?

E Drink a beverage from a restaurant in a plastic cup?

F Drink a beverage from a fast food restaurant?

Personal care information

G Take a shower or a bath?

H Use liquid shower gel or hand soap when bathing or showering?

I Apply hand or body lotion?

J Apply deodorant?

K Wash your hands in only water?

L Wash your hands with soap and water?

M Get a cash register receipt?

N Get a receipt when you used cash?

O Get a receipt when you used a credit card?

P Get a receipt from a department store?

Q Get a receipt at the gas pump?

R Get a receipt at the grocery store?

S Use cash to purchase your gas and got a receipt?

T Use cash to purchase your groceries and got a receipt?

Dental

U Have you had a sealant applied to your teeth in the past three months?

V Have you been to the dentist in the past three months?

Smoking

W Do you smoke

X How many cigarettes a day do you smoke?

Specify the number if known: __________

Y How many cigarettes are you around a day due to second-hand smoke?

Additional personal care information

Z Did you take a bath or shower this morning?

AA Men: Did you apply cologne in the past two days?

AB Men: Did you apply aftershave in the past two days?

AC Women: Are you currently menstruating?

AD Women: First day of your last menstrual flow?

AE Women: How long are your typical menstrual cycles?

AF Women: What type of birth control do you currently use?

AG Women: Tampons or pads?

AH Women: Tampon or pad+brand+if tampon cardboard or plastic

How many times in the past 48 hours did you:

AI Place your receipt in your purse or wallet?

AJ Place it in your pocket?

AK Place it in your car after refueling?

AL Place it in your car after getting items from a drive-through window?

AM Place receipts in a designated spot to view what you spent later?

AN Touch a receipt when you clean out a car?

AO Touch receipts when you compare them to your checkbook entries?

AP Touch receipts when you check your debit card transactions?

AQ Use receipts to check your credit card transactions?

AR What is your occupation+how often do you handle receipts?
